# Oxyanion Removal from Impaired Water by Donnan Dialysis Plug Flow Contactors

**DOI:** 10.3390/membranes13110856

**Published:** 2023-10-26

**Authors:** Shalom Fox, Kristina Stadnik, Amit K. Thakur, Lior Farkash, Zeev Ronen, Yoram Oren, Jack Gilron

**Affiliations:** Zuckerberg Institute for Water Research, Blaustein Institutes of Desert Research, Ben-Gurion University of the Negev, Midreshet Ben-Gurion 84990, Israel; foxshalom@gmail.com (S.F.); kristina179@walla.com (K.S.); amitjshandilya@gmail.com (A.K.T.); liorfarkash27@gmail.com (L.F.); zeevrone@bgu.ac.il (Z.R.); yoramo@bgu.ac.il (Y.O.)

**Keywords:** Donnan dialysis, plug flow contactor, perchlorate removal, nitrate removal, groundwater remediation

## Abstract

In the last twenty-five years, extensive work has been done on ion exchange membrane bioreactors (IEMB) combining Donnan dialysis and anaerobic reduction to remove trace oxyanions (e.g., perchlorate, nitrate, chlorate, arsenate) from contaminated water sources. Most studies used Donnan dialysis contactors with high recirculation rates on the feed side, so under continuous operation, the effective concentration on the feed side of the membrane is the same as the exit concentration (CSTR mode). We have built, characterized, and modelled a plug flow Donnan dialysis contactor (PFR) that maximizes concentration on the feed side and operated it on feed solutions spiked with perchlorate and nitrate ion using ACS and PCA-100 anion exchange membranes. At identical feed inlet concentrations with the ACS membrane, membrane area loading rates are three-fold greater, and fluxes are more than double in the PFR contactor than in the CSTR contactor. A model based on the nonlinear adsorption of perchlorate in ACS membrane correctly predicted the trace ion concentration as a function of space-time in experiments with ACS. For PCA membrane, a linear flux dependence on feed concentration correctly described trace ion feed concentration as a function of space-time. Anion permeability for PCA-100 was high enough that the overall mass transfer was affected by the film boundary layer resistance. These results provide a basis for efficiently scaling up Donnan dialysis contactors and incorporating them in full-scale IEMB setups.

## 1. Introduction

There is increasing stress on world water resources as population grows along with climate-induced droughts and anthropogenic contamination of existing water sources (e.g., explosives manufacture, fracking, excessive fertilizer use). One group of contaminants frequently found in groundwater contains oxyanions such as nitrate, perchlorate, chromate, and selenate. Arsenate is a naturally occurring contaminant in groundwater sources frequently found in the Indian sub-continent, Africa, and in some European countries.

The presence and distribution of oxyanions such as perchlorate and chlorate in the environment are becoming a critical international water quality concern [1,2]. Perchlorate is a common contaminant in many parts of the world, especially in the US and China, where anthropogenic activities are responsible for widespread perchlorate contamination of drinking water, surface water, groundwater, and soil [3]. It is manufactured as perchloric acid and inorganic perchlorate salts for various applications. These have been significantly introduced into the environment in the form of disinfectants, bleaching agents, blasting agents, herbicides, and rocket propellants [2,3,4]. Exposure to ammonium perchlorate has been reported to lead to hypothyroidism [4], which can impact normal growth and development in fetuses, infants, and young children [5]. The perchlorate anion (ClO_4_^−^) as a stable, negatively charged ion appearing in salts that are highly water-soluble [6] is an extremely mobile compound in aquatic environments. Since perchlorate is not absorbed by natural sediments [7], it spreads rapidly and easily contaminates surface water [2] as well as the saturated porous zone of the sub-aquifer [8]. Spontaneous environmental degradation of perchlorate does not occur because perchlorate has a high activation energy [9], resulting in a significant kinetic barrier for its reduction [10]. The mobility and stability of perchlorate, combined with the fact that it is easily absorbed and accumulated by different crops, makes this chemical extremely hazardous [11]. A maximum concentration level (MCL) of 15 [μg L^−1^] is the target concentration level for drinking water recommended by the American environmental protection agency [12], and the State of California has set a maximum contaminant level (MCL) of 6 [μg L^−1^] in drinking water [13].

The concentrations found in the environment range between a few µg L^−1^ and thousands of mg L^−1^ [3,4,14]. In the US, the highest reported concentrations of perchlorate were 811 µg L^−1^ in drinking water, 3700 mg L^−1^ in ground water, 120 mg L^−1^ in surface water, and 2000 mg kg^−1^ in soil [12]. In Israel, perchlorate pollution was found in the coastal aquifer underlying the industrial area of Ramat Ha’Sharon and Tel-Aviv and a military industry area near Rehovot. Perchlorate concentrations of up to 250 [mg L^−1^] were found in the groundwater and up to 1200 [mg per kg of sediment] were found in the unsaturated zone below wastewater holding ponds [15]. Perchlorate concentrations of 450 mg L^−1^ were found in the groundwater under the pilot area in Ramat Hasharon [16]. The estimated volume of perchlorate-contaminated water is about 1 billion m^3^ in Ramat Ha’Sharon groundwater [16].

While most nitrate contamination comes from excessive fertilizer use and feedlot operations, it is also often found at the same sites as perchlorate contamination. This is so because perchlorate explosives are mostly in the form of ammonium salts (NH_4_ClO_4_) that can oxidize to nitrates, while nitrates themselves are also used in explosives. The major effect of nitrate in humans, especially in young infants, is its involvement in the oxidation of normal hemoglobin (Hb) to metHb, which is unable to transport oxygen to the tissues. As a result, blue baby syndrome (=metHb) is the main concern of nitrate pollution in drinking water [17]. In addition, nitrate in surface waters contributes significantly to eutrophication. The MCL for nitrate is 40 [mg L^−1^].

The presence of the above contaminants results in the closing of wells that otherwise could supply badly needed drinking water. In light of the above, methods for efficient treatment to remove the contamination have been extensively studied. Existing means for the treatment of water contaminated with oxyanions include ion exchange and adsorption, reverse osmosis, chemical reduction, bio-degradation, electrochemical reduction, bio-electrochemical reduction, and hybrid technologies [3,9,14,18,19]. Both IX and RO have the challenge of dealing with regeneration solutions, or brine concentrates with high levels of contaminating anions, whereas IX has an additional issue of competition by common anions for the sites intended for lower-concentration contaminants [14].

Microbial reduction is a commonly used approach, converting perchlorates and chlorates to chloride and nitrate to nitrogen [9]. While nitrate has a similar redox potential to perchlorate (+1.25 V), its chemical structure makes the nitrogen atom more available for reduction, with lower activation energy (47.18 [kJ mol^−1^]) than perchlorate [3,19]. The conventional bioreactors include fluidized-bed biofilm (FBB), using either sand or GAC as the support media [20,21], or fixed-bed biofilm reactor (FBR) with acetate, acetic acid, molasses, or ethanol as the electron donor [14,22]. These are suitable for the treatment of highly concentrated waste streams [3].

Standalone microbial remediation has rarely been used directly in drinking water treatment systems because of public health issues with the high-quality standards demanded for drinking water. The possibility of secondary contamination of treated water with microbial cells and their metabolic by-products is of real public concern. In addition, organic residuals can increase demand for chlorine for disinfection, which can lead to the formation of unacceptable disinfection by-products (DBP) in the treated water [3]. However, the recent application of FBB technology (combined with filtration and disinfection) for the treatment of perchlorate-polluted groundwater resulted in effluents which met the drinking water standards [21].

Due to the above drawbacks of the common biological treatments for drinking water, integrated technologies have been sought, combining both removal and biological reduction. These technologies include H_2_-based hollow-fiber membrane biofilm reactors [23,24,25], bioelectrical reactors [26,27], and ion exchange membrane bioreactor (IEMB) [28]. H_2_-based hollow-fiber membrane biofilm reactors have been tested in a pilot scale to remove the low level of perchlorate (154 ± 5 μg/L) to 9 ug/L, and a full-scale MBfR system for treatment of nitrate (ARoNite^TM^) in drinking water was permitted to run at the Cucamonga Valley Water District [25].

This group has been active in studying IEMB [29,30,31,32], first pioneered by the group at the New University of Lisbon [28,33,34,35,36,37,38,39,40] headed by Prof. Joao Crespo. Ion exchange membrane bioreactors use a Donnan dialysis contactor composed of an anion exchange membrane with a concentrated solution of chloride as the driving ion on the receiving side to pull oxyanions over from the feed side. Microbial bioreduction occurs on the receiving side of the membrane isolated from the feed solution. Thus, no secondary contamination of the treated feed stream occurs.

Except for our recent work with spiral wound anion exchange contactors [31,32], almost all studies of both IEMB and Donnan contactors have been carried out in slit flow cells with high recycle rates either in the continuous mode with high recycle rates [28,29,30,33,34,35,36,37,38,39,40]) or Donnan dialysis in the batch mode [41,42,43]. The disadvantage of continuous operation of a Donnan contactor with a high recycle rate (analogous to continuous stirred tank reactors—CSTR) is that the feed concentration next to the feed side of the membrane is the same as that of the exit concentration, and since flux is a function of concentration, the flux is lower than it otherwise would be. Applying a plug flow contacting mode for continuous operation through a contactor with a long path length allows the concentration on the feed side to be maintained at its maximum value and decrease gradually along the membrane as the oxyanions are removed. In this respect, it carries out in space what the batch reactor does in time. This is what we did in the spiral wound contactors used with a 6 m path length. However, verification of the plug flow models was limited by only having access to the inlet and exit concentration of the contactor.

In the present study, we investigated a Donnan dialysis PFR contactor with sampling ports periodically located along a 6 m feed-side path. The PFR performance is compared to CSTR-type slit cells of similar flow cross-section. Further, we verified the models describing Donnan dialysis in a PFR contacting mode similar to those previously described in the literature for batch mode operation.

## 2. Theory

Several models have been suggested for formulating the Donnan dialysis transport as a function of concentration and membrane properties. One main approach [44,45] proposed the Nernst–Planck equation as a basis for modelling transport and assuming zero convective flux and zero swelling pressure. The group of Hasson et al. [41,42,43]—extensively developed this approach in batch operation. They showed that the Donnan dialysis flux for two solutions with different anions, A and B, separated by an anion exchange membrane could be expressed by [41]:

(1) $$J_{A}=P_{S,A}\left[\frac{C_{A,1}}{C_{+,1}}-\frac{C_{A,2}}{C_{+,2}}\right]=-J_{B}=P_{S,B}\left[\frac{C_{B,2}}{C_{+,2}}-\frac{C_{B,1}}{C_{+,1}}\right]$$

Generalizing from Equations (17) and (18) in [41]:

(2) $$\begin{array}{l} \frac{1}{P_{S,A}}=\frac{1}{P_{S0,A}}+\frac{\delta_{1}}{D_{A,1}C_{+,1}}+\frac{\delta_{2}}{D_{A,2},C_{+,2}}=\frac{1}{P_{S0,A}}+\frac{1}{k_{d1}C_{+,1}}+\frac{1}{k_{d2}C_{+,2}}\\ \mathrm{where}:\;\;\frac{1}{P_{S0,A}}=\frac{L_{m}}{X_{m}D_{A,m}} \end{array}$$

Additionally, for similar mass transfer conditions on either side of the membrane, this reduces to the expression from Equation (17) in [41]:

(3) $$\frac{1}{P_{S,A}}=\frac{1}{P_{S0,A}}+\frac{1}{k_{D,A}}\left(\frac{1}{C_{+,1}}+\frac{1}{C_{+,2}}\right)$$

$P_{S0,A}$ is, in fact, the intrinsic permeability of the membrane to component A. In our case, the module geometry and hydrodynamics are different on either side of the membrane, so we must use Equation (2) as is. The right-hand side of Equation (2) states that the resistance to Donnan transport is the sum of the membrane and liquid-side mass transfer resistance, respectively. In our system, in which driving ion concentration in the receiving side is much higher than in the feed side and in which the removal of the contaminant ion does not exceed 60–70%, the second term on the right-hand side of Equation (1) can be neglected, namely:

(4) $$\frac{C_{A,2}}{C_{+,2}}<<\frac{C_{A,1}}{C_{+,1}}$$

resulting in:

(5) $$J_{A}\approx P_{S,\;A}\frac{C_{A,1}}{C_{+,1}}=aC_{TA,1}\;\mathrm{where}:\;\;a=\frac{P_{S,\;A}}{C_{+,1}}$$

In describing the change in the concentration along the flow path of a plug flow contactor with a slit flow channel of width ω and a channel height h, the following mass balance can be made:

(6) $$Q\frac{dC}{dx}=-J\omega$$

If J is a linear function of the concentration (Equation (4)) and coefficient a is the constant, then the following integrated solution is obtained with the boundary condition, *C* = *C*_0_ at *x* = 0:

(7) $$C=C_{0}\exp\left[\frac{-a\omega x}{Q}\right]$$

Which can be written in terms of space-time, τ, as:

(8) $$C=C_{0}\exp\left[\frac{-ax}{hu}\right]=C_{0}\exp\left[\frac{-a\tau }{h}\right]$$

where *u* is the average linear feed velocity (=*Q*/*ωh*) in the slit channel. Therefore, the logarithm of the normalized concentration C/$C_{0}$ is expected to decline linearly with the slope *a*/*h*. This was indeed validated in the PFR system equipped with the PCA-100 membrane (see Section 4.3).

On the other hand, the results obtained for the ACS membrane showed flux was not linear in the feed-side concentration of perchlorate (see Section 4.1). An alternative model will be provided in Section 4.2.

## 3. Materials and Methods

### 3.1. Materials

Sodium perchlorate, sodium nitrate, and sodium chloride (all analytical grade) were provided by Sigma-Aldrich. The membranes used were ACS monovalent selective anion exchange membrane from Astom Corporation, Japan, and PCA-100, from PC-Cell GmbH, Germany. The properties of the two types of membranes featured in this study as determined from small sample swatches are shown in Table 1.

### 3.2. Analytical Methods

Anion concentrations in the water compartment were determined using an ion chromatograph by Dionex ICS 5000 (Sunnyvale, CA, USA) with an Ion Pac^TM^ AG19 column (detection limit of ±0.01 mg L^−1^) (Thermo Fisher Scientific, Waltham, MA, USA), with 60 mM KOH eluent for perchlorate and 20 mM KOH for nitrate, chloride, and sulfate. Perchlorate from the receiving-side solution was analyzed by a perchlorate-specific ion combination electrode (SIE) (Cole Parmer, Vernon Hills, IL, USA) detection limit of ±0.5 mg L^−1^). Nitrate samples from the receiving side were analyzed by a UV second derivative method (Ferree & Shannon 2001) in a Shimadzu UV-1800 spectrophotometer (the detection limit was ±0.1 mg N L^−1^) (Tokyo, Japan).

### 3.3. Experimental Setup

#### 3.3.1. Feed and Bleed CSTR Donnan Dialysis Contactor

The membrane module was composed of Plexiglas with two identical flow channels with dimensions of 0.3 cm height, 25 cm length, and 1.5 cm width, which makes an active membrane area of 37.5 cm^2^. The feed and receiving compartments were connected to 0.1–0.25 L and 0.5–0.6 L reservoir tanks, respectively, fed with trace oxyanion contaminants and 0.1 M NaCl, respectively, by two peristaltic pumps–Cole Parmer MasterFlex 7550-60 Computerized Drive pump and Gilson (Dunstable, UK) Miniplus 3 Peristaltic Pump). In the case of perchlorate experiments with the ACS membrane, the feed and receiving solutions were as shown in Table S1 in the Supplementary Materials, in which the main ion composition mimicked groundwater on the feed side and biomedia on the receiving side. The temperature of the solutions was maintained at 25 °C by a bath chiller (WBL-100, MRC, Holon, Israel).

**Figure 1:**
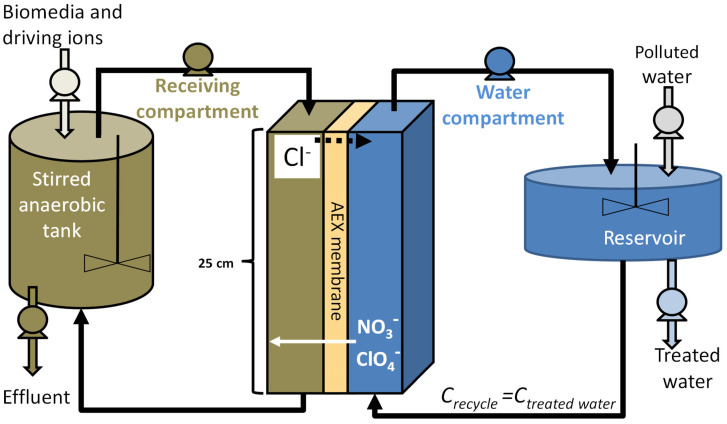
Setup for the operation of an IEMB operating with a Donnan exchange contactor in a CSTR feed-and-bleed mode. After [28].

Both compartments operated in feed-and-bleed mode as continuously stirred tank reactors (CSTR) (see Figure 1), with a re-circulating rate of 1.2–1.6 [L min^−1^] by two gear pumps (DGMO9, Fluid-o-Tech, Milan, Italy). The flow rate in both flow channels ensured a turbulent flow regime (Reynolds number of 2400-2777). More details can be found in [29,46].

A summary of the experimental conditions can be found in Table 2. The flowsheet for this setup is shown in Figure 1.

#### 3.3.2. The Plug Flow Contactor

**Figure 2:**
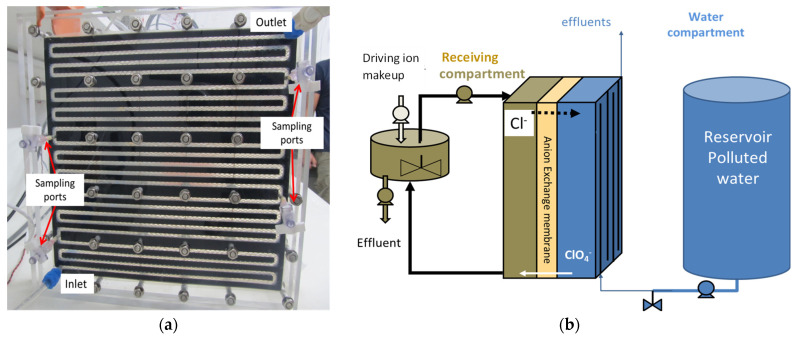
Experimental setup for PFR Donnan dialysis contactor. (**a**) Feed side of the flow cell with sampling ports along the path length. (**b**) Schematic flowsheet showing incorporation of Donnan dialysis contactor with the feed side flowing in the tortuous path on the right side of the contactor and the receiving side on the left side with the anion exchange membrane between the two sides.

The plug flow Donnan dialysis (PFR) contactor comprised a feed solution flow channel and receiver solution flow channel with the anion exchange membrane placed between the feed and receiving solution compartments. With overall contactor dimensions of 25 cm by 30 cm, the effective membrane area was 300 cm^2^, lining a tortuous feed channel of 588 cm total length. Six sampling ports were positioned along the feed water compartment channel as follows: at the inlet and outlet and four intermediate sampling ports at a distance of 102, 230, 358, and 486 cm from the inlet (see Figure 2a). The end plates of the PFR contactor were constructed from Plexiglas, while a 3D printer (Stratasys, Rehovot, Israel) was used for fabricating the spacer channel in both the feed and receiver compartments. The spacer channels were made of nylon with a silicone rubber coating for sealing. In the first version, the feed spacer flow channel contained a turbulence-promoting net with a filament thickness of 0.05 cm, a total thickness of 0.13 cm, and 60° angle between the filaments. An ACS membrane was used with this first version. Due to the mechanical fragility of the spacer on the feed side, a later version (version 2) of the feed-side spacer was 3D printed with 0.35 cm thickness instead of 0.13 cm thickness, and this version was used with the PCA-100 anion exchange membrane. In both versions, the flow channel on the receiving solution side was 0.5 cm high. The feed and receiving compartment channel dimensions of the two versions are summarized in Table 3.

The receiver solution compartment was operated similarly to the CSTR receiver compartment, where the receiving solution reservoir was positioned upstream of the receiver side of the contactor and the liquid was circulated through the receiving compartment channel at a flow rate of 0.84 ± 0.050 L min^−1^ (Table 4), promoting strong turbulent regime along the channel and uniform composition on the receiver side (Figure 2b). Considering that and that C_2+_ >> C_1+_, the last term on the right-hand side of Equation (2) can be dropped as negligible relative to the other terms.

The feed water was delivered into the water compartment using a peristaltic pump by Master Flex Digital Standard driver model no. 7523-70 with an Easy-Load^TM^ head and a 16 L/S Pharmed tube, with an in-line pulsation dampener to have a steadier flow. The contaminated water passed through the water side channel once. Temperature of the feed water was regulated at 22 °C (±1 °C) and monitored; the axial pressure and the flow rate as well as the temperature for both compartments were continuously monitored.

The electrical conductivity (EC) changes, pH, and the circulation flow rate of the receiving solution were continuously monitored. Other parameters such as water effluent pH, EC, and flow rates were monitored manually. The operating data (flow rates, pH, pressures) were logged on a Novus Field-logger data logger (Novus, Canoas, Brazil).

The conditions for the PFR experiments are summarized in Table 4.

The receiving side refers to fresh solution injection and bleed flow rate since being operated in the feed-and-bleed mode.

The Donnan dialysis experiments were carried out for two inlet concentrations of perchlorate, 30 and 100 [mg L^−1^], with no other ions added. The receiving compartment solution was 0.1M of NaCl in DI water. Each inlet concentration was studied under the following volumetric flow rates: 11, 15, 22, and 28 [mL min^−1^]. The perchlorate concentration profile along the channel was characterized for each flow rate. The results were normalized to space-time, which is the linear velocity of the liquid in the channel divided by the distance from inlet allowing us to compare the results from each different experiment run. When the thicker spacer was used (version 2 cell), the volumetric flow rates on the feed side were 18, 32, 52, and 72 [mL min^−1^] to achieve similar linear velocities and space-time values to those of the thinner spacer. The version 2 cell was fed on the feed side with solutions that were either perchlorate (100 mg/L), nitrate (200 mg/L), or mixtures of perchlorate and nitrate as the sodium salts and a background concentration of 5 meq/L of sodium chloride. The receiving side was the same as above.

The loading rate of trace anion into the feed side [meq h^−1^ m^−2^] is given by:

(9) $$Loading\;Rate=\frac{Q_{f}C_{TA,1,in}}{A}$$

The flux of trace ion across the membrane is given by:

(10) $$\begin{array}{ll} J_{TA} & =\frac{Q_{f}\left(C_{TA,1,in}-C_{TA,1,out}\right)}{A}=\left(1-\frac{C_{TA,1,out}}{C_{TA,1,in}}\right)\cdot \frac{Q_{f}C_{TA,1,in}}{A}\\ & =\left(\mathrm{Fractional}\;\mathrm{removal}\right)\left(Loading\right) \end{array}$$

## 4. Results

### 4.1. CSTR Operation and Adsorption Isotherms

**Figure 3:**
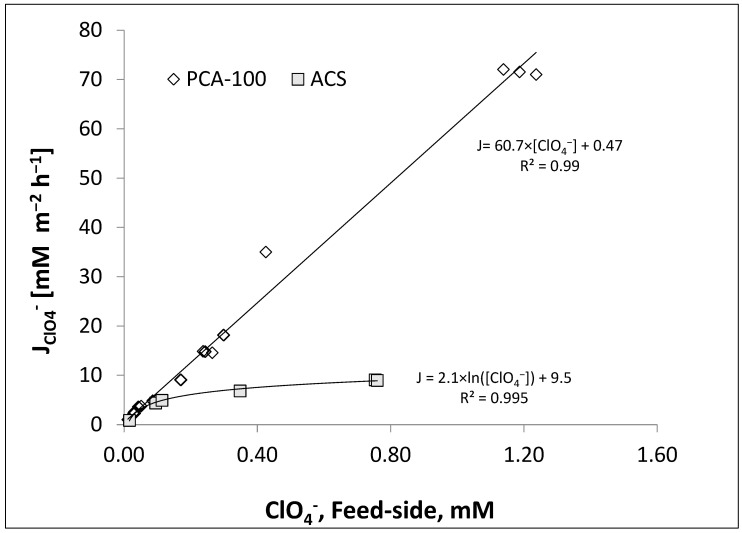
Perchlorate flux across the membrane as a function of perchlorate concentration on the feed side of the membrane (same as feed-side exit concentration) in the CSTR contacting pattern. Fresh feed flow rate 19.2 mL/h introduced into feed line recycling at 1600 mL/min.

The oxyanion fluxes obtained by running a CSTR-type membrane contactor with two different membranes are shown in Figure 3. For the ACS membrane, perchlorate flux deviates from linear dependence on feed-side concentration, approaching a plateau. On the other hand, perchlorate flux for the PCA100 membrane remains linear throughout the tested feed concentration range (0.1–1.2 mM), validating the assumption resulting in Equation (4).

**Figure 4:**
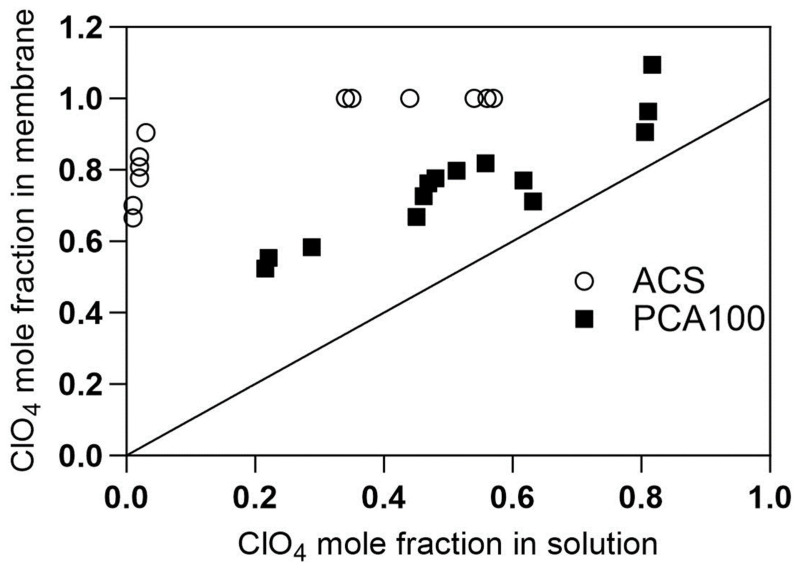
Adsorption isotherms for perchlorate ion in different anion exchange membranes.

**Figure 5:**
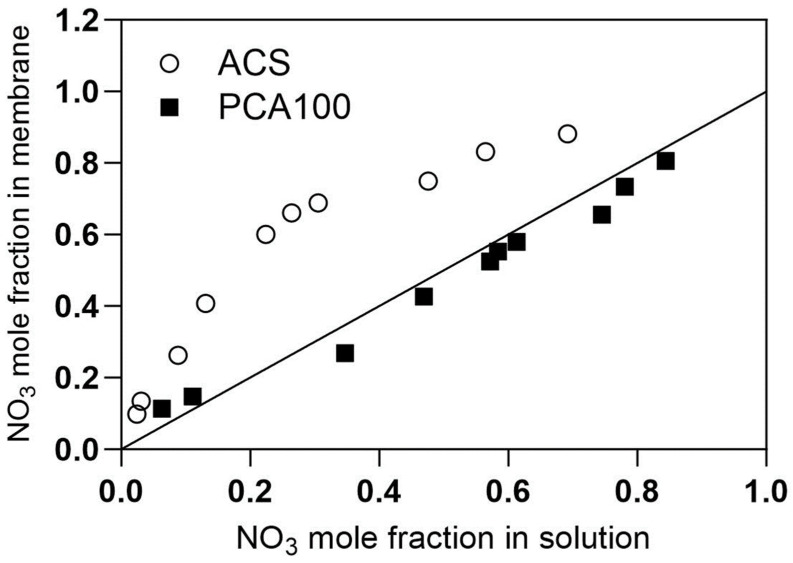
Adsorption isotherms for nitrate in contact with ACS membrane and PCA-100 anion exchange membrane. Diagonal line shows the non-selective curve (equivalent fraction in membrane = equivalent fraction in solution).

The differing flux-concentration dependencies can be understood based on the adsorption isotherms for perchlorate in the presence of chloride for the two membranes shown in Figure 4. The perchlorate selectivity of the ACS membrane is pronounced, reaching saturation of ion exchange sites when the equivalent fraction of perchlorate in the external solution is less than 10%. By contrast, the equivalent fraction of perchlorate in the PCA-100 membrane is only slightly greater than its equivalent fraction in the solution, and its equivalent fraction in the solution must reach at least 50% equivalent fraction in the solution to occupy 80% of the fixed charge sites in the membrane. While not as pronounced as the case with perchlorate, similar behavior can be seen with nitrate (Figure 5), in which there is a preference for nitrate over chloride in ACS membrane, whereas no significant selectivity is evident for nitrate over chloride in the PCA-100 membrane.

The perchlorate flux-concentration behavior of the ACS membrane can be explained by using the Blaedel model for Donnan dialysis of a trace component [47], as extended by Velizarov et al. [28,35,38]. They obtained the following set of equations for a trace anion TA with chloride as the driving ion:

(11) $$J=\frac{\frac{{\bar{C}}_{Cl,1}}{C_{Cl,1}}C_{TA,1}-\frac{{\bar{C}}_{Cl,2}}{C_{Cl,2}}C_{TA,2}}{\frac{L_{m}}{P_{m}}+\frac{\delta_{1}{\bar{C}}_{Cl,1}}{D\;C_{Cl,1}}+\frac{\delta_{2}{\bar{C}}_{Cl,2}}{D\;C_{Cl,2}}}$$

If the target ion is a trace and the membrane is NOT selective to it, then the concentration of the driving ion in the membrane will be nearly the same throughout the membrane interior and equal to the membrane fixed charge capacity *X_m_*. In this case, Equation (11) can be expressed as:

(12) $$J=\frac{\frac{X_{m}}{C_{Cl,1}}C_{TA,1}-\frac{X_{m}}{C_{Cl,2}}C_{TA,2}}{\frac{L_{m}}{P_{m}}+\frac{\delta_{1}X_{m}}{D\;C_{Cl,1}}+\frac{\delta_{2}X_{m}}{D\;C_{Cl,2}}}=\frac{\frac{C_{TA,1}}{C_{Cl,1}}-\frac{C_{TA,2}}{C_{Cl,2}}}{\frac{L_{m}}{P_{m}X_{m}}+\frac{\delta_{1}}{D\;C_{Cl,1}}+\frac{\delta_{2}}{D\;C_{Cl,2}}}$$

In the case of ACS anion exchange membrane, the membrane has such a high affinity for perchlorate ions that it occupies a significant fraction of the fixed charge sites of the membrane even when it is only a small equivalent fraction of the external solution. Thus, ${\bar{C}}_{Cl,1}$ cannot be considered constant but may become significantly less than the ion exchange capacity and changes as the target ion concentration in the feed solution changes. As a result, the full flux expression of Equation (11) must be retained.

The regression of the perchlorate isotherm (Figure 4) can provide an expression for the chloride concentration in the membrane as follows:

(13) $${\bar{C}}_{Cl,1}=X_{m}\left(1-\left(0.0686\ln\left(y_{ClO_{4},1}\right)+1.0592\right)\right)\;\mathrm{where},\;y_{ClO_{4},1}\equiv \frac{C_{ClO_{4},1}}{C_{ClO_{4},1}+C_{Cl,1}}$$

*y*_*ClO*4,1_ is the equivalent fraction of perchlorate in the feed solution and *X_m_* is the fixed charge concentration in the membrane. This helps explain the logarithmic dependence of flux on concentration for the ACS membrane (Figure 3).

### 4.2. PFR Results for ACS Membrane

In Table 5, a comparison of the results from the CSTR and PFR contactors is presented for ACS membrane on a feed containing perchlorate. The PFR contactor is operated in one case at the same perchlorate loading (F/A) as the CSTR (in bold in the fourth row of data in first two columns) and with the same inlet concentration, C_ClO4,in_, (in bold in the first row of data in last two columns) in the other case.

At the same loading (F/A, as mmol ClO_4_^−^/h/m^2^), the PFR contactor is more efficient, generating a higher flux (6.4 mmol-h^−1^-m^−2^ vs. 5.5 mmol-h^−1^-m^−2^) and a greater removal extent (94.2% vs. 86.4%) even though the log-mean feed-side perchlorate concentration is lower. At the same inlet concentration under high loading conditions, the PFR has more than three times the membrane-specific loading rate (F/A) and more than double the flux. This can be understood as stemming from a higher volumetric flow rate and the higher log mean feed-side concentration of perchlorate in the PFR as compared to the CSTR. These comparisons highlight the higher efficiency of the PFR contacting mode over the CSTR mode.

**Figure 6:**
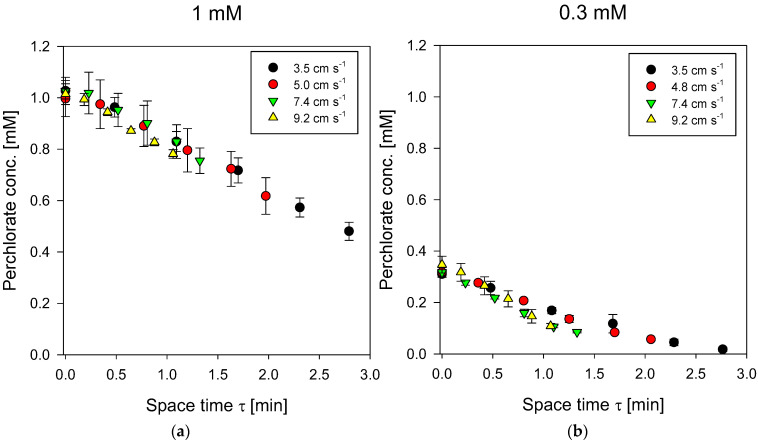
Perchlorate concentration as a function of space-time in PFR contactor with channel height of 0.13 cm. Inlet perchlorate concentration on feed side was (**a**) 1 mM (left) or (**b**) 0.3 mM (right). Different symbols represent different average linear velocities in the feed channel.

Figure 6 displays the perchlorate concentration profiles as a function of distance from the PFR contactor inlet for various flow rates. By dividing the distance along the flow reactor by the average feed velocity, the space-times for each data point could be generated, showing all the data in a consolidated graph. A careful examination of the data at 1 mM entrance concentration (Figure 6a) and 0.3 mM entrance concentration (Figure 6b) shows that there is little impact of feed flow rate when plotted in terms of space-time. This implies membrane mass transfer resistance is dominant compared to solution boundary layer mass transfer resistance (see Equations (2) and (11)).

**Figure 7:**
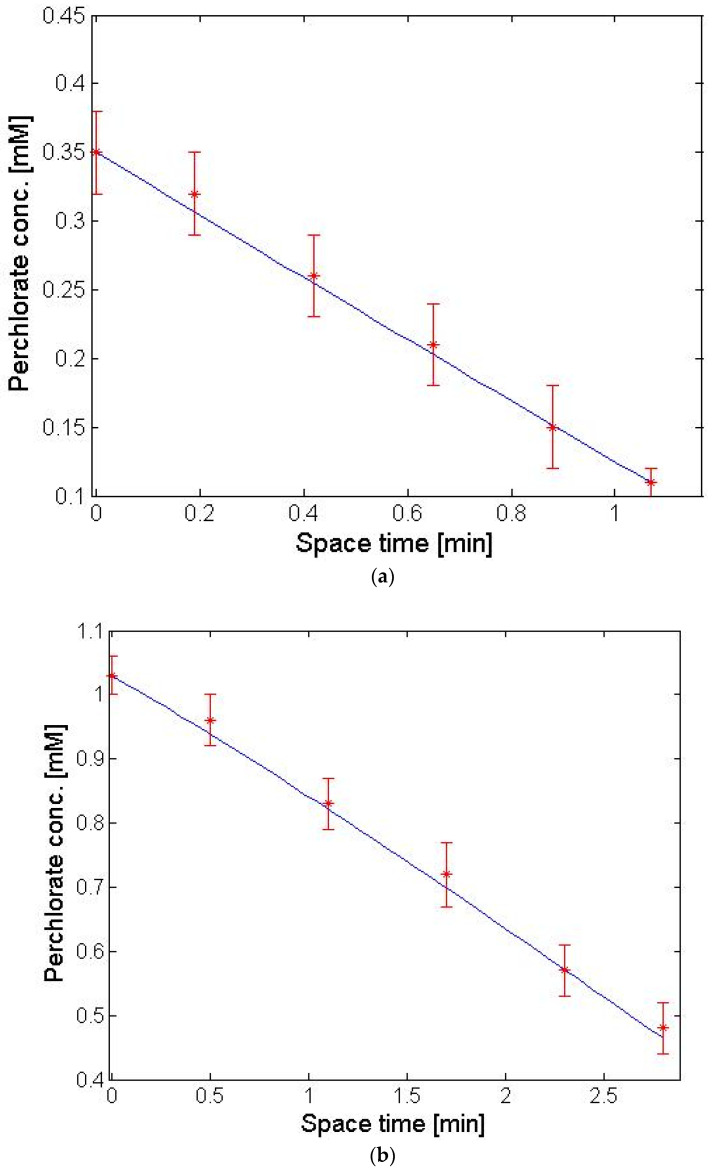
Model prediction (solid line) and actual results for PFR contactor (symbols) with (**a**) feed rate of 15 L/d containing 1 mM perchlorate and 3.2 mM chloride and (**b**) feed rate of 40 L/d containing 0.35 mM perchlorate and 3.2 mM chloride.

Furthermore, we see the slope of the concentration space-time curve is almost not affected by the concentration. This reflects the very shallow slope of the perchlorate flux vs. concentration curve for ACS in Figure 3. The isotherm for the ACS membrane, as presented in Equation (13), expresses the chloride concentration in the membrane on the feed side of the AEM as a function of feed perchlorate solution concentration next to the membrane. Therefore, to model the flux in a PFR contactor equipped with ACS membrane, we must use Equation (11) for J including the explicit dependence of ${\bar{C}}_{Cl,1}$ on the concentration of perchlorate ion expressed in Equation (13) and numerically integrate Equation (6) (see details in Supplementary Materials). This can then generate the concentration profile as a function of space-time in the PFR contactor. This was done assuming the film-side mass coefficients were given by the mass transfer correlation of Schock and Miquel [48] for a spacer filled channel and the membrane permeability for perchlorate was treated as a fitting parameter. The calculations were realized in MATLAB and the results are displayed in Figure 7. It is evident that the simulated perchlorate removal curve as a function of space-time reproduces the experimental result well.

### 4.3. PFR Results for PCA-100 Membrane

**Figure 8:**
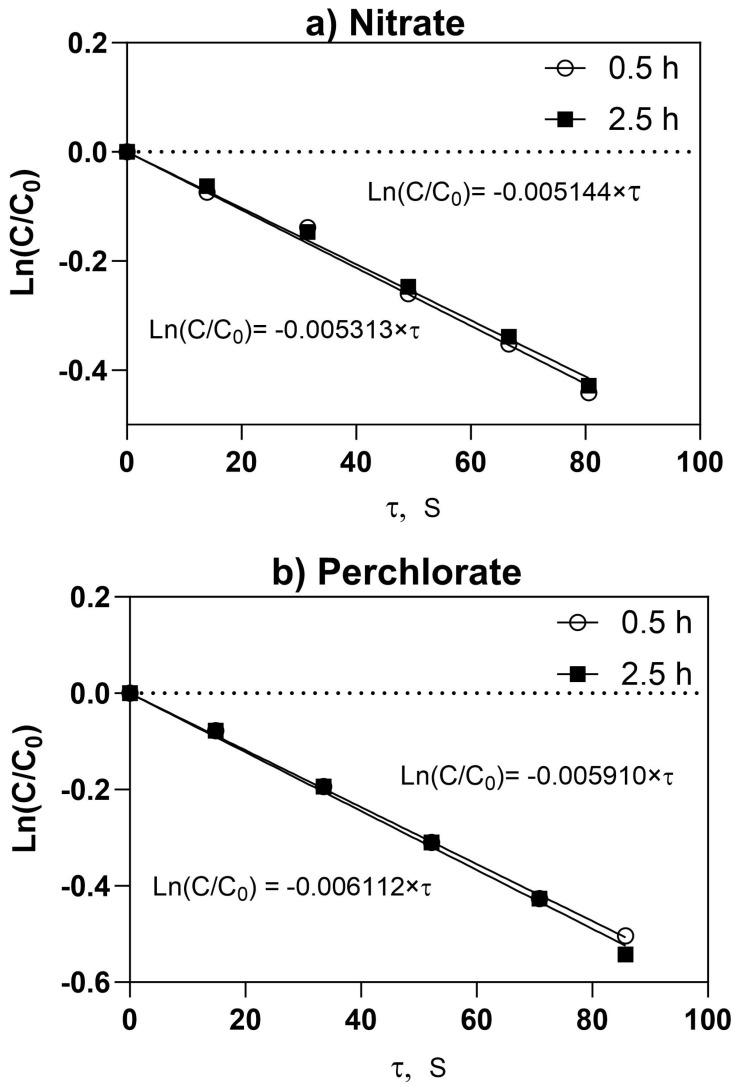
Logarithm of normalized feed-side concentration dependence on space-time, τ, in PFR contactor equipped with PCA-100 membrane. (**a**) Nitrate (C_o_~210 mg/L) (**b**) perchlorate (C_o_~100 mg/L) feed flow rate 72 mL/min. Hours refer to sampling time during steady state run of the PFR.

Concentration dependencies for nitrate and perchlorate on the space-time for the PFR contactor operated with the PCA-100 membrane at different flow rates are shown in Figures S1 and S2 respectively. Based on Equation (8), the data can be recast into a linear form by plotting *ln* of the local concentration normalized by the entrance concentration (C/Co) as a function of space-time, τ. As can be seen in Figure 8 for both nitrate (Figure 8a) and the perchlorate concentration (Figure 8b), this results in a linear dependence as expected for a linear flux vs. feed concentration dependence at any point along the flow path of the PFR contactor. Based on Equation (8), the slope $m_{TA}$ of the straight line for the particular trace anion (TA) is given by:

(14) $$m_{TA}=\frac{-a_{TA}}{h}$$

where $a_{TA}$ is proportional to the membrane permeability to the target anion and is defined in Equation (5), while h is the feed flow channel height.

**Figure 9:**
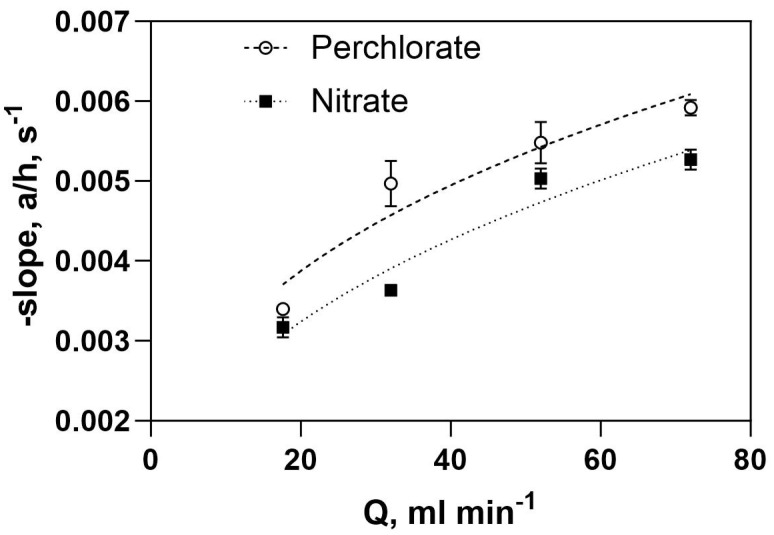
Dependence of PFR overall mass transfer slope on feed flow rate.

By carrying out similar regressions as used in Figure 8 according to Equation (8), we can generate a table of the slopes of these plots for all flow conditions for the two oxyanions (Table 6). Since the slope is inversely proportional to the overall total mass transfer resistance (see Equations (2) and (3)) and the slope is larger for perchlorate compared to that for nitrate, it can be argued that the membrane ion permeation resistance is somewhat lower for perchlorate than it is for nitrate at the same flowrate. The results of these experiments were then reinterpreted using Equation (5) and plotted for the slope as a function of feed flow rate (Figure 9). In the case of both perchlorate and nitrate (Figure 9), the absolute value of the slope increases with increasing flow rate, nearly reaching a plateau. Unlike the case in the ACS membrane, this implies that the perchlorate and nitrate membrane mass transfer resistances (see first term in denominator in RHS of Equations (4) and (7)) are low enough that the feed-side liquid film mass transfer resistance becomes significant. This can be seen by inspecting the slopes for perchlorate flux vs. feed concentration curves with both membranes (Figure 3), where a higher slope can be interpreted as lower membrane mass transfer resistance for the PCA membrane.

**Figure 10:**
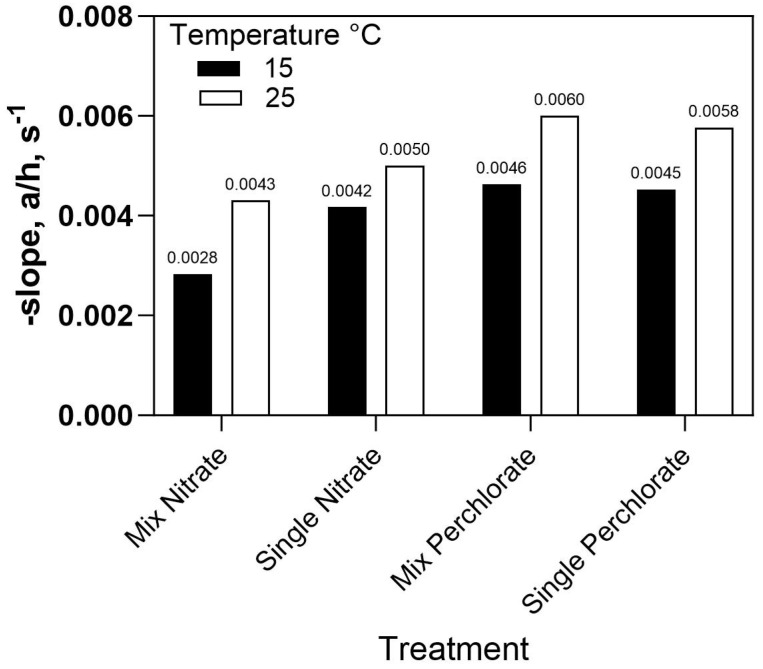
Slope of the concentration decline curve as a function of temperature and presence of competing oxyanion for perchlorate and nitrate.

The effect of temperature on oxyanion fluxes for the PCA-100 membrane is shown in Figure 10. Temperature has a clear effect on trace anion flux across the membrane for both perchlorate and nitrate, as we would expect for any activated process like diffusion through a membrane. For the same membrane, when comparing the flux of nitrate and perchlorate as pure solutions or as present in mixtures, we see that nitrate flux is significantly affected by the presence of perchlorate, but perchlorate is only slightly affected by the presence of nitrate. This can be understood from the adsorption isotherms for PCA-100 still being more selective for perchlorate (Figure 4) than nitrate (Figure 5).

## 5. Conclusions

Operating a Donnan dialysis contactor in the plug flow mode provides significantly higher perchlorate fluxes than operating a contactor in the feed-and-bleed mode with high recycle, which approximates CSTR mode. PFR contactors fitted with ACS and PCA-100 anion exchange membranes fed with 1 mmol/L of perchlorate achieved perchlorate loadings as high as 21.9 mmol/h/m^2^ and 145 mmol/h/m^2^, respectively. In contrast, a CSTR contactor had a perchlorate loading of only 6.4 mmol/h/m^2^ using the ACS membrane fed with 1 mmol/L perchlorate.

The PFR contactor performance with perchlorate ion in the feed was successfully modelled for the ACS membrane using the Blaedel–Velizarov equation combined with the empirical perchlorate adsorption isotherm for ACS membrane. In contrast, a first-order flux-concentration dependence was demonstrated for PFR equipped with PCA-100 membrane consistent with CSTR results for this membrane treating perchlorate spiked feed. Similarly, the Donnan dialysis studies of nitrate ion in the PFR equipped with PCA-100 membrane also demonstrated a first-order dependence of flux on the nitrate concentration in the whole flow length of the PFR. The overall mass transfer coefficients for perchlorate were slightly higher than those for nitrate, indicating a somewhat higher membrane permeability for the former anion. In mixtures of the two anions, perchlorate transport was unaffected by the presence of the nitrate but nitrate transport was slightly reduced by the presence of perchlorate, as reflected in the empirical mass transfer coefficients. Membrane mass transfer coefficients increased with temperature, as would be expected.

These results clearly point the way to using PFR-type contactors to scale up the Donnan dialysis contactors and the IEMB systems that incorporate them, as was confirmed by our recent work using spiral wound Donnan contactors with a similar 6 m path length [31,32].

## Figures and Tables

**Table 1:** Water uptake (WU) and ion exchange capacity (IEC) measured for the membranes used in this study.

Membrane	Water Uptake (WU) (%)	IEC (meq g^−1^), Dry Weight
ACS-Neosepta-sample 1	24.17	1.91
ACS-Neosepta-sample 2	24.70	1.99
PCA-100-sample 1	29.62	1.99
PCA-100-sample 2	29.47	2.07

**Table 2:** Operating conditions in the CSTR Donnan dialysis contactor.

Operating Parameter	Membrane Tested			
	ACS		PCA-100	
	Feed side	Receiving side	Feed Side	Receiving side
1. Polluted feed flow rate, mL/h	19.2–25.2	13.8–144	22–82	122–358
2. Recycle rate, mL/min	1600	1200–1550	1500	1500
3. Reservoir HRT, h	5.2–14	0.75–6.25	4.4–16.2	4.9–17
4. Contactor residence time, min	0.007	0.007–0.009	0.0075	0.0075
5. Perchlorate experiments,				
[Cl^−^]in, meq/L	3.52	109.4	5	100
[ClO_4_^−^]in, meq/L	0.2–2.5	0	1.2–16.8	0
6. Nitrate experiments,				
[Cl^−^]in, meq/L	3.52	109.4	5	100
[NO_3_^−^]in, meq/L	0.01–0.92	0	0.73–10.9	0
7. Perchlorate/Nitrate,				
[Cl^−^]in, meq/L	3.52	109.4	7–7.5	100
[NO_3_^−^]in, meq/L	0.01–1.2	0	1.2–16.8	0.0
[ClO_4_^−^]in, meq/L	1	0	0.73–10.9	0.0

**Table 3:** Donnan dialysis/IEMB PFR contactor cell dimensions.

	Length [cm]	Width [cm]	Depth [cm]	Compartment Volume [cm^3^]	Sampling Ports
Feed compartment	588	0.5			6
Version 1 ACS membrane			0.13	38.2	
Version 2 PC-100 membrane			0.35	103	
Receiver compartment	588	0.5	0.5	3500 ^a^	2

^a^ Includes external stirred tank reservoir provided in recycling.

**Table 4:** Experimental operating conditions of the PFR Donnan dialysis contactor.

Operating Parameter	Membrane Used			
	PFR VS 1		PFR VS 2	
	ACS		PCA-100	
	Feed side	Receiving side	Feed Side	Receiving side
1. Flow rate ^a^, mL/min	10–30	10–25	18–72	47–50
2. Recycle rate, mL/min	0	850	0	850
3. Contactor residence time, min	1.0–2.9	0.16	0.4–4.9	0.16
4. Perchlorate experiments,				
[Cl^−^]in, meq/L	0.15–10	100	5	100
[ClO_4_^−^]in, meq/L	0.3, 1.0	0	1.0	0
5. Nitrate experiments,				
[Cl^−^]in, meq/L			5	100
[NO_3_^−^]in, meq/L			3.2	0
6. Perchlorate/Nitrate, meq/L				
[Cl^−^]in, meq/L			7–7.5	100
[NO_3_^−^]in, meq/L			3.2	0.0
[ClO_4_^−^]in, meq/L			1.0	0.0

^a^ Includes external stirred tank reservoir provided in recycling.

**Table 5:** Comparison of CSTR and PFR reactor modes for perchlorate removal in Donnan dialysis using ACS membrane samples.

	Same Loading and Different Inlet Concentrations		Same Inlet Concentrations and Different Loading	
CONFIGURATION	PFR	CSTR	PFR	CSTR
Inlet conc (mM)	0.31	0.99	**1.03**	**0.99**
Outlet conc (mM) (=average conc for CSTR)	0.018	0.136	0.480	0.136
Log mean [mM]	0.102	0.136	0.719	0.136
F/A_m_ [mM m^−2^ h^−1^] normalized loading	**6.7**	**6.4**	21.9	6.4
Avg channel J specific flux [mM m^−2^ h^−1^]	6.4	5.5	11.8	5.5
Removal efficiency for water comp (load in–load out)/load in	94.4%	86.2%	53.2%	86.2%

**Table 6:** Mass transfer slopes for PFR contactor fitted with PCA membrane.

	1000 × -m_TA_. s^−1^	
Q (mL/min)	NO_3_^−^	ClO_4_^−^
17.6	3.10	3.40
32	3.63	4.97
52	4.78	5.48
72	5.07	5.82

## Data Availability

The data presented in this study are available on request from the corresponding author.

## Supplementary Materials

The following supporting information can be downloaded at: https://www.mdpi.com/article/10.3390/membranes13110856/s1, Figure S1: Feed-side nitrate concentration in PFR as function of space time, Figure S2: Feed-side perchlorate concentration in PFR as function of space time, Figure S3: Plot of the membrane resistance M(C) as a function of feed side perchlorate concentration, Table S1: Water and bio-compartment basic ion composition, Table S2: Physical quantities used in calculation of concentration profiles of PFR equipped with ACS membrane.

## Nomenclature

a_TA_	Overall target anion permeability, defined in Equation (5)
A_m_	Membrane area, m^2^
1/*P_So_*	Membrane mass transfer resistance, h-m^2^/meq
1/Ps	Overall mass transfer coefficient, h-m^2^/meq
C_+_	Concentration of cations, meq/L
C_Cl,1_	Concentration of chloride in feed solution, meq/L
C_Cl,2_	Concentration of chloride in receiver solution, meq/L
$\bar{C}$	Concentration in the membrane, meq/L
d_h_	Hydraulic diameter, cm
D_c_	Diffusivity coefficient of target ion, m^2^/s
$\bar{D}$	Diffusivity coefficient of ion in the membrane, m^2^/s
F	Target anion loading rate, mmol/h
h	Channel height, cm
J	Ion flux from water compartment to bio-compartment, meq/m^2^-h
k_d_	Film-side mass transfer coefficient, m/h
L_m_	Membrane thickness, cm
P_so_	Membrane permeability for anion, meq/m^2^-h
Q	Flow rate, cm^3^/s
Re	Reynolds number
S	Effective membrane area, cm^2^
Sc	Schmidt number
*u*	Flow velocity, cm/s
V_1_	Feed volume, cm^3^
V_2_	Receiver volume, cm^3^
x	Distance from feed channel entrance, cm
X_m_	Fixed charge density in anion exchange membrane, meq/L
*y*	Equivalent fraction in solution
*y_m_*	Equivalent fraction in membrane
*ω*	Slit flow channel width, cm
α	Reynolds number exponent
τ	Space-time, s
Subscripts	
1	Feed-solution side
2	Receiving-solution side
A	Contaminant ion
B	Driving ion
f	Feed
m	Pertaining to anion exchange membrane
o	Initial value
TA	Target anion
