# Supplementary material for: Oxyanion Removal from Impaired Water by Donnan Dialysis Plug Flow Contactors
Source: Membranes (Basel). 2023 Oct 26;13(11):856. doi: 10.3390/membranes13110856 (PMC10673252; doi:10.3390/membranes13110856)
Supplement: Supplementary file 1 [file membranes-13-00856-s001.zip › membranes-2539841-supplementary.pdf]

Supplementary information

1. DATA

The solution compositions for the feed and receiving solutions during CSTR-type experiments using perchlorate and ACS membrane are provided in table S-1. The ACS membrane was initially chosen since it had been used by the group of Crespo and Velizarov and co-workers [27,34-41] in developing the IEMB process. Given its relatively low perchlorate permeability, the present authors then chose PCA-100 from PC-Cell (whose ED equipment had been used by us in previous studies). PCA-100 according to the PC-Cell data sheet has higher anion permeability as it is used for retaining low molecular weight organic acids.

Table S1: Water and bio compartment basic Ion composition

	**Ion**	**water compartment chemical composition [mN]**	**bio compartment chemical composition [mN]**
	**H^+**	-	16.2
	**Na^+**	4	100.3
	**K^+**	0.1	19.2
	**Ca^{+2}**	3.6	-
	**Mg^{+2}**	0.8	0.8
	**NH_4^+**	-	4.4
cations	**Sum**	**8.5**	**140.9**
	**Cl^-**	3.3	104.6
	**SO_4^{-2}**	1.2	0.8
	**HCO_3^-**	3.2	-
	**NO_3^-**	0.9	-
	**PO_4^{-3}**	-	35.5
anions	**sum**	**8.5**	**140.9**
	**pH**	7.5	7.2
	**EC**	0.83	12.4

The compilation of the PFR experiments run with PCA membrane at different flow rates is provided for nitrate (Figure S1) and perchlorate (Figure S2) as the trace anion.

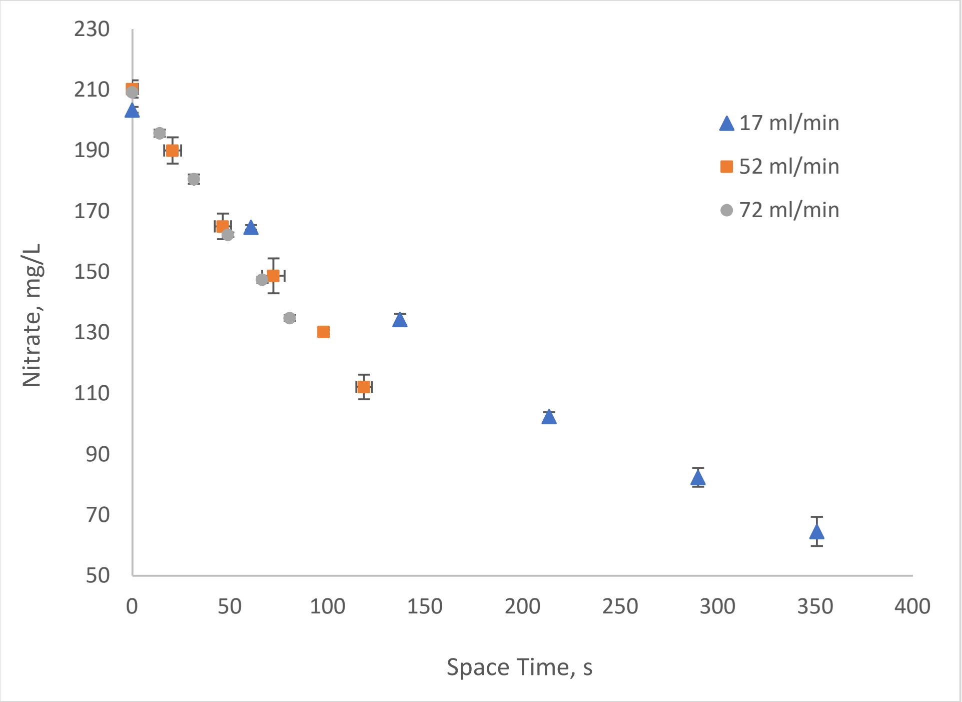

Figure S1 – Feed-side nitrate concentration in PFR as function of space time

:

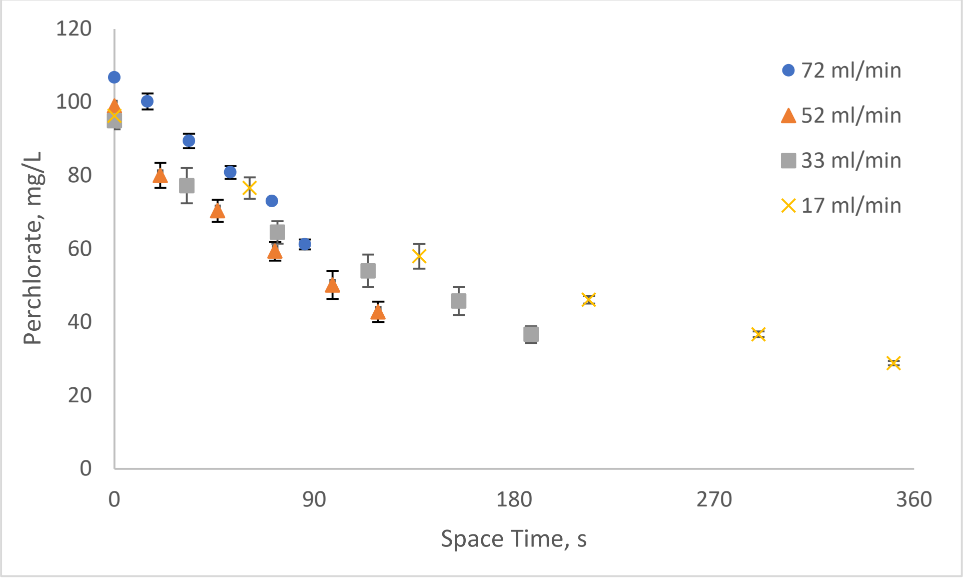

Figure S2 - Feed-side nitrate concentration in PFR as function of space time

2. Modelling PFR contactor equipped with ACS membrane

We start with equation 11 from the paper:

$$J = \frac{\frac{\overline{C}_{Cl,1}}{C_{Cl,1}} \cdot C_{ClO_4^-,1} - \frac{\overline{C}_{Cl,2}}{C_{Cl,2}} \cdot C_{ClO_4^-,2}}{\frac{L_m}{P_m} + \frac{\delta_1 \overline{C}_{Cl,1}}{DC_{Cl,1}} + \frac{\delta_2 \overline{C}_{Cl,2}}{DC_{Cl,2}}}$$

We define the following intermediate functions (Velizarov et al., 2002):

[S1] $$EDF = \frac{\overline{C}_{Cl,1}}{C_{Cl,1}} \cdot C_{ClO_4,1} - \frac{\overline{C}_{Cl,2}}{C_{Cl,2}} \cdot C_{ClO_4,2}$$

$$B = \frac{\delta_1 \overline{C}_{Cl,1}}{DC_{Cl,s,1}} + \frac{\delta_2 \overline{C}_{Cl,2}}{DC_{Cl,s,2}}$$

We then regress on Lm/Pm from the data from the CSTR experiment for perchlorate:

[S2] $$\frac{L_m}{P_m} \equiv M(c) = \frac{EDF}{J_i} - B$$

The values of $\overline{C}_{Cl,1}$ and $\overline{C}_{Cl,2}$ needed to evaluate EDF and B are evaluated from the CSTR stream composition data using equation 13 in the paper. The mass transfer correlations to evaluate the mass transfer resistances on the feed and receiving side respectively are given by (Schock & Miquel, 1987) for the low velocity flow on feed side and for turbulent flow by Mulder on the receiving side:

[S3] feed $$\boxed{S_h = 0.065 S_c^{0.25} \mathrm{Re}^{0.875}}$$

Receiving side $$\boxed{S_h = 0.04 S_c^{0.33} \mathrm{Re}^{0.75}}$$

The resulting dependence M(C) on feed perchlorate concentration is plotted in figure S3.

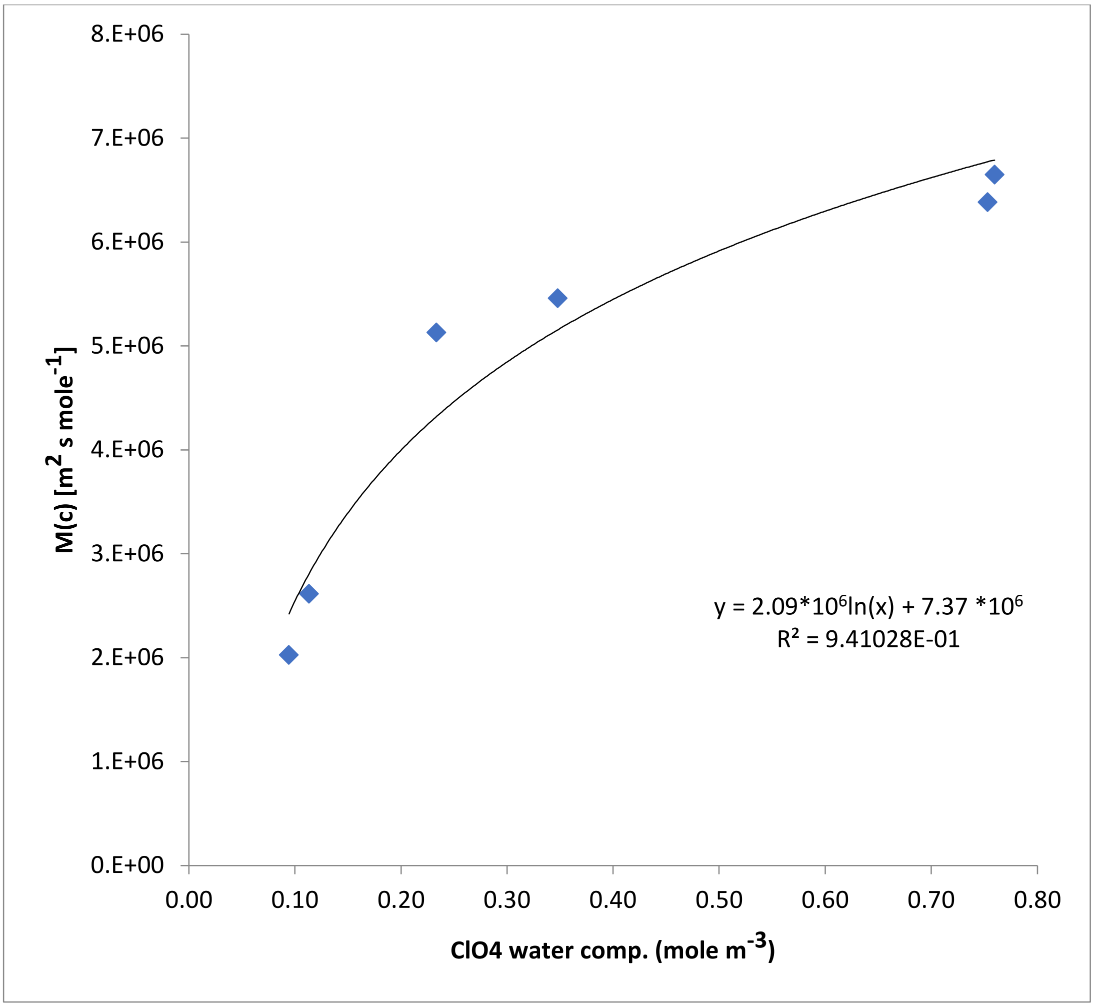

Figure S3: Plot of the membrane resistance M(C) as a function of feed side perchlorate concentration.

The regression equation on the membrane resistance is given by;

$$M(c) = a_1 \ln(C_{i,1}) + b_1$$
$$a_1 = 2.09331 \times 10^6$$
$$b_1 = 7.36549 \times 10^6 \quad [S4]$$

The concentration of chloride ion in the membrane is given by the from the empirical isotherm found for perchlorate in the ACS membrane (equation 13 of the paper):

$$\overline{C}_{Cl,i} = X_m\left(1-\left(0.0686\ln\left(y_{ClO_4,i}\right)+1.0592\right)\right) \text{ where } y_{ClO_4,i} \equiv \frac{C_{ClO_4,i}}{C_{ClO_4,i}+C_{Cl,i}} \quad [13]$$

Where i = 1 (feed side) or 2 (receiver side).

Substituting these expressions into equation [S1] we have:

[S5]

$$EDF = \frac{X_m\left(1-\left(0.0686\ln\left(y_{ClO_4,1}\right)+1.0592\right)\right)}{C_{Cl,1}}\cdot C_{ClO_4,1} - \frac{X_m\left(1-\left(0.0686\ln\left(y_{ClO_4,2}\right)+1.0592\right)\right)}{C_{Cl,2}}\cdot C_{ClO_4,2}$$

$$B = \frac{\delta_1 X_m\left(1-\left(0.0686\ln\left(y_{ClO4,1}\right)+1.0592\right)\right)}{D_{i,W}C_{Cl^-,1}} + \frac{\delta_2 X_m\left(1-\left(0.0686\ln\left(y_{ClO4,2}\right)+1.0592\right)\right)}{D_{i,W}C_{Cl^-,2}}$$

Because of the high concentration of chloride on the receiving side and the high recirculation rate on the receiving side, we can take the concentration of Ccl,2 equal to its exit concentration. On the feed side we use the electrical balance requirement that total anion concentration must be constant to write the following balance equation connecting the change in perchlorate and chloride concentrations:

[S6]

$$\begin{aligned} &C_{Cl^-,1} + C_{ClO_4^-,1} = C^o_{Cl^-,1} + C^o_{ClO_4^-,1} \\ &\Rightarrow C_{Cl^-,1} = C^o_{Cl^-,1} + \left(C^o_{ClO_4^-,1} - C_{ClO_4^-,1}\right) \end{aligned}$$

Where the superscript, 0, refers to entrance concentrations. Substituting the expression for $C_{Cl^-,1}$ from equation S6 into the expressions for EDF and B in equation S5, the expression for the flux becomes:

[S7]

$$J = \frac{\frac{C_{ClO_4^-,1}}{C^o_{Cl^-,1}+\left(C^o_{ClO_4^-,1}-C_{ClO_4^-,1}\right)} X_m\left(1-\left(0.0686\ln\left(x_{i,1}\right)+1.0592\right)\right) - \frac{C_{ClO_4^-,2}}{C_{Cl^-,2}} X_m\left(1-\left(0.0686\ln\left(x_{i,2}\right)+1.0592\right)\right)}{\left(a_1\ln(C_{ClO_4^-,1}) + b_1 + \frac{\delta_1 X_m\left(1-\left(0.0686\ln\left(x_{i,1}\right)+1.0592\right)\right)}{D_{i,W}\left(C^o_{Cl^-,1}+\left(C^o_{ClO_4^-,1}-C_{ClO_4^-,1}\right)\right)} + \frac{\delta_2 X_m\left(1-\left(0.0686\ln\left(x_{i,2}\right)+1.0592\right)\right)}{D_{i,W}C_{Cl^-,2}}\right)}$$

This can be substituted into equation 6 of the paper (mass balance on a differential section of the feed side of the plug flow reactor) to give :

[S8]

$$\begin{aligned} &Q\frac{dC}{dx} = -J\omega \Rightarrow \frac{dC}{d\tau} = -\frac{J}{h} \\ &\text{where } Q = u\omega h \quad \text{and} \quad dx = u\,d\tau \end{aligned}$$

Equations S7 and S8 were integrated using MATLAB. The values for the different physical quantities used in the calculation are given in table S2.

Table S2: Physical quantities used in calculation of concentration profiles of PFR equipped with ACS membrane

h_{feed}	0.00117	m
$h_{receiving}$	0.005	m
ω_{feed}	0.005	m
$w_{receiving}$	0.005	m
dh, receiving	0.00667	m
$u_{receiving}$	0.49	m/s
υ	$1 \cdot 10^{-6}$	m^2/s
D(perchlorate)	$1.79 \cdot 10^{-9}$	m^2/s
Xm	1830	$equiv/m^3$
